# Supplementary material for: Uncertainty in ensembles of global biodiversity scenarios
Source: Nat Commun. 2019 Mar 29;10:1446. doi: 10.1038/s41467-019-09519-w (PMC6441032; doi:10.1038/s41467-019-09519-w)
Supplement: Supplementary file 1 — Supplementary Information [file 41467_2019_9519_MOESM1_ESM.pdf]

Uncertainty in ensembles of global biodiversity scenarios

W. Thuiller, M. Guéguen, J. Renaud, D. N. Karger and N. E. Zimmermann

SUPPLEMENTARY INFORMATION

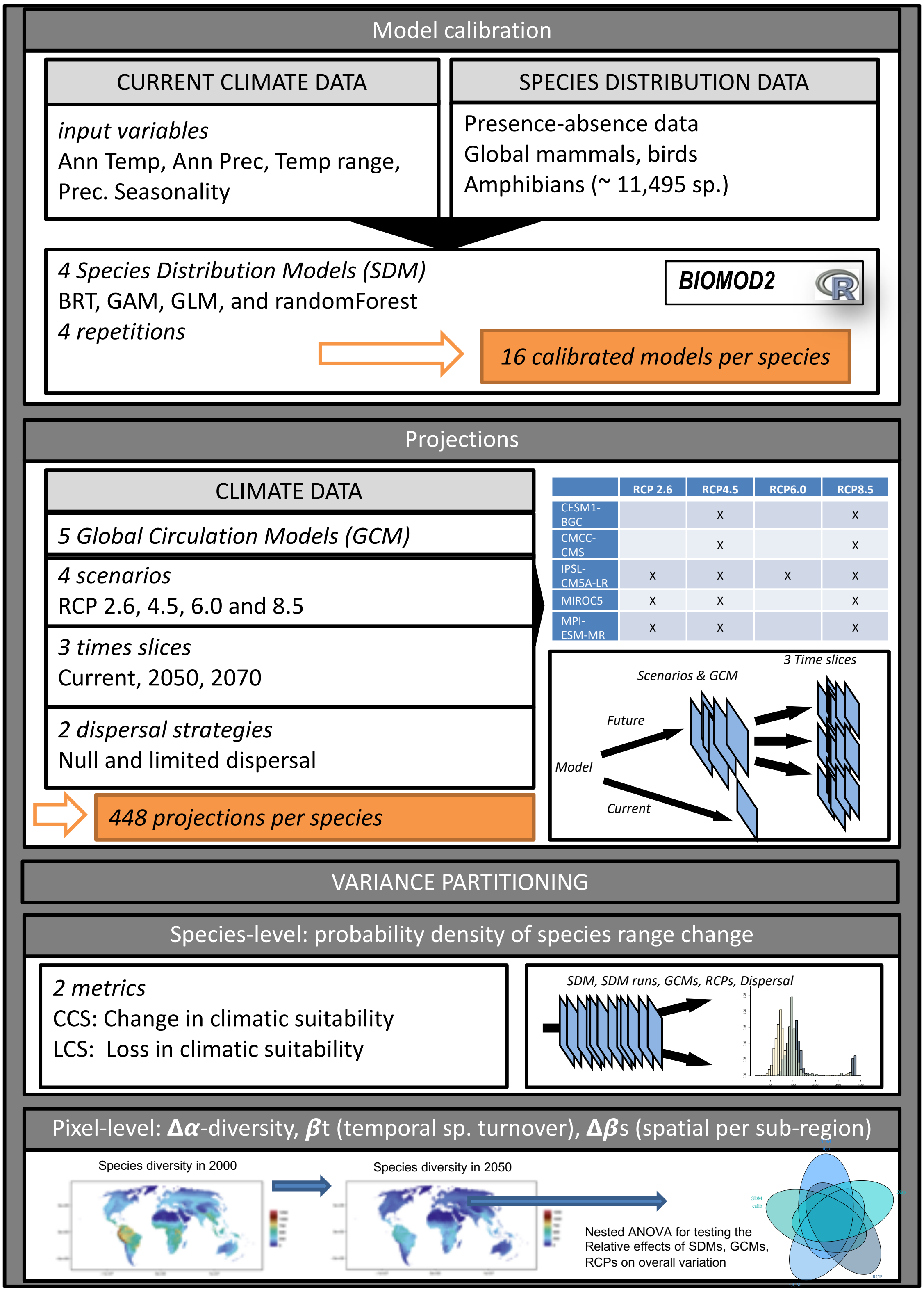

Supplementary Figure 1. Workflow diagram depicting the different steps of the analysis performed in this study. Different steps are the data formatting, calibration of the species distribution models (SDMs), forecasting of future species distributions under several combinations of global circulation models (GCMs) and representative concentration pathways (RCPs), calculation of sensitivity metrics and variance partitioning of the uncertainty.

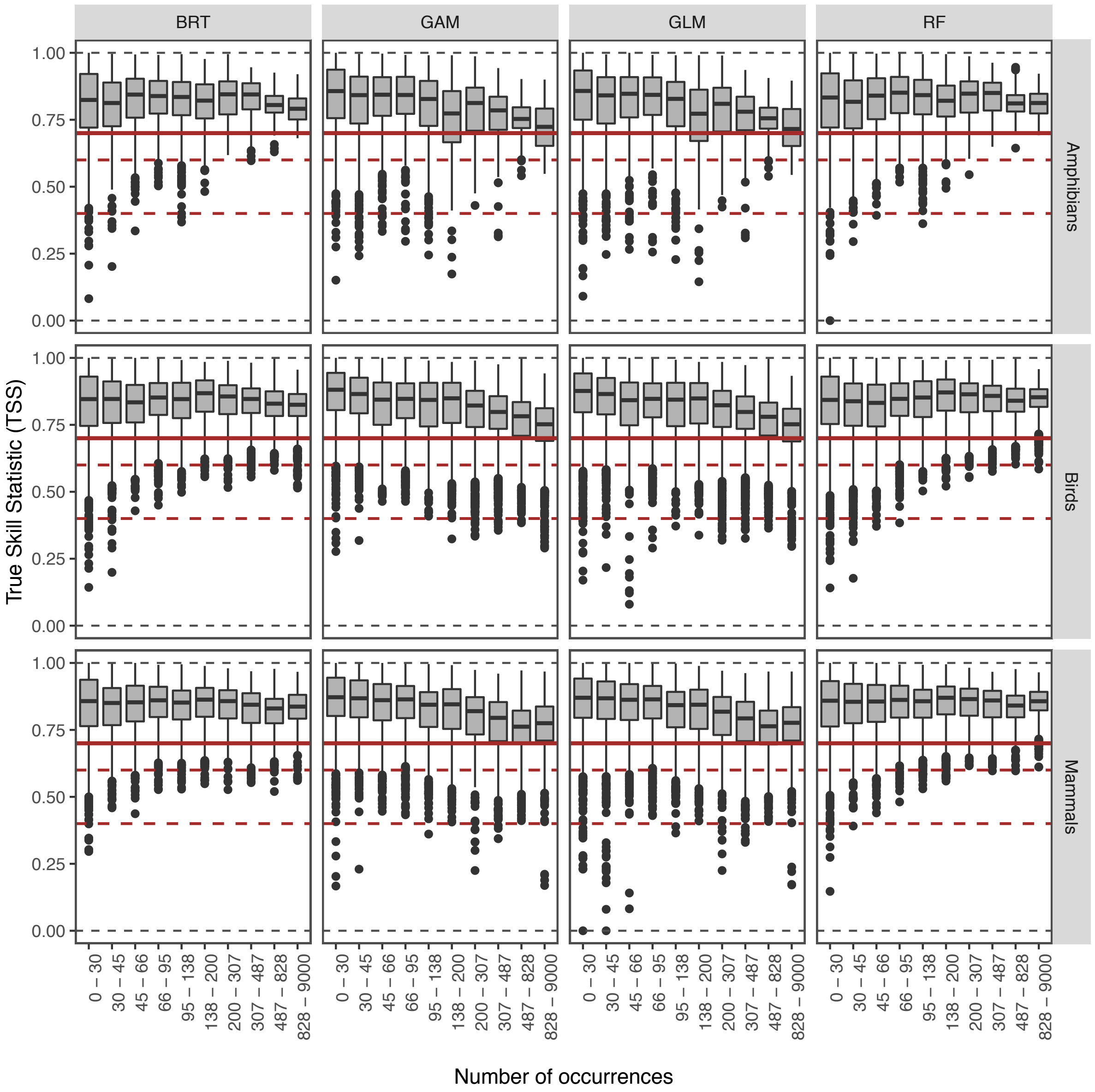

Supplementary Figure 2. Predictive performance of the four species distribution models to predict the distribution of the three vertebrate species groups. BRT, GAM, GLM and RF represent boosted regression tree, generalized additive model, generalized linear model and random forest. RUN1 to RUN4 represents the four cross-validation runs based on repeated splitting of the training data. The variation in TSS represents the different TSS values obtained for each of the 11,495 species in the cross-validation test. The red lines correspond to TSS thresholds of 0.4 (short dash), 0.6 (long dash) and 0.7 (solid), above which we retained models in ensembles for assessing the influence of predictive quality on the overall uncertainty of species-based metrics. The highest threshold of 0.7 was kept in all further analyses.

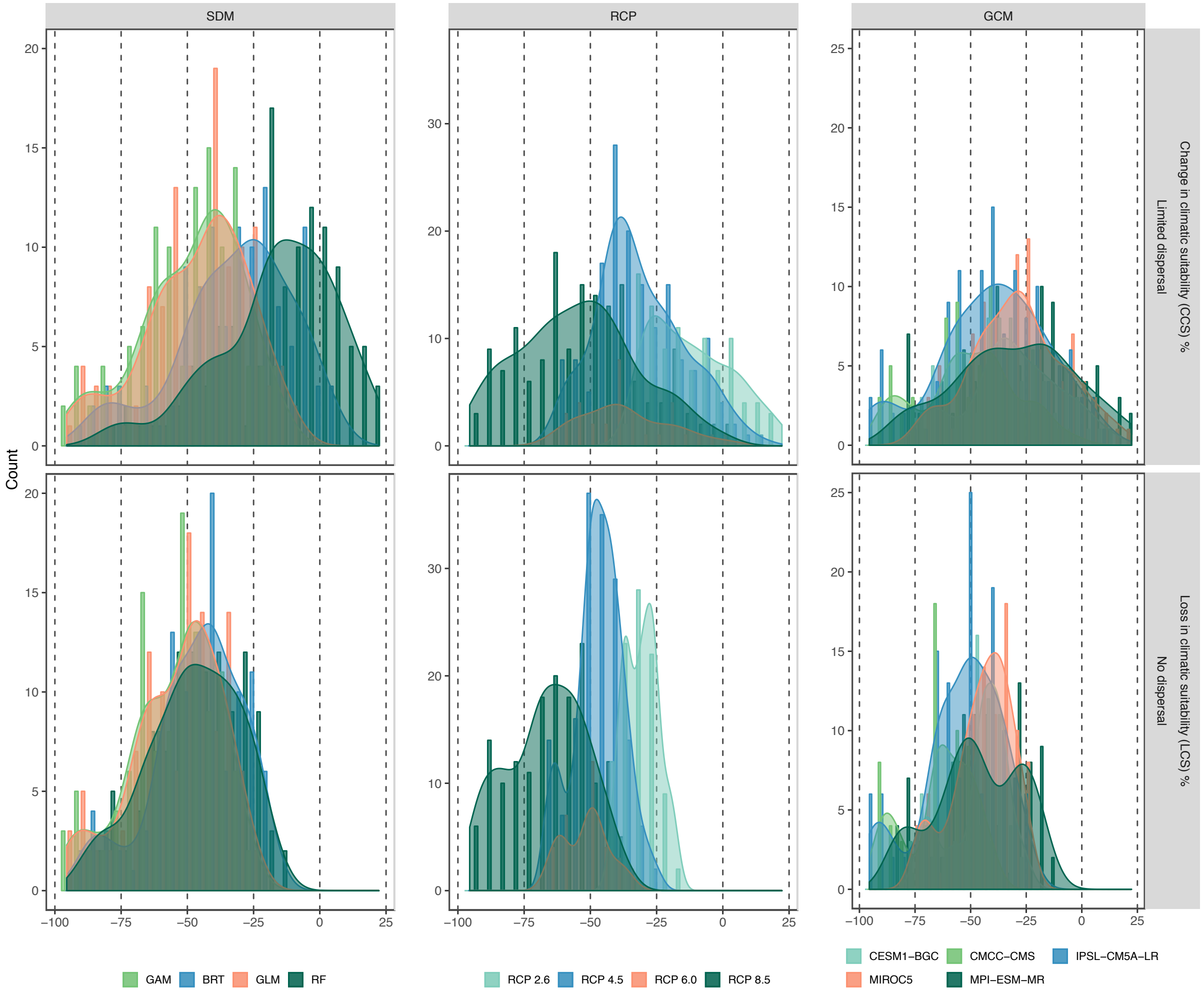

Supplementary Figure 3. Probability density function as a function of changes in suitable climates (upper panel) and loss in suitable climates (lower panel) for the bearded woodpecker (*Dendropicos namaquus*). For change in suitable climate, the x-axis goes from -100% (i.e. extinction) to +25% (range extension by 25%). For loss in suitable climate, this goes from -100% (complete loss) to 0% (no loss of currently suitable climate space). From left to right, is represented the variation due to SDMs, RCPs and GCMs on the total variation.

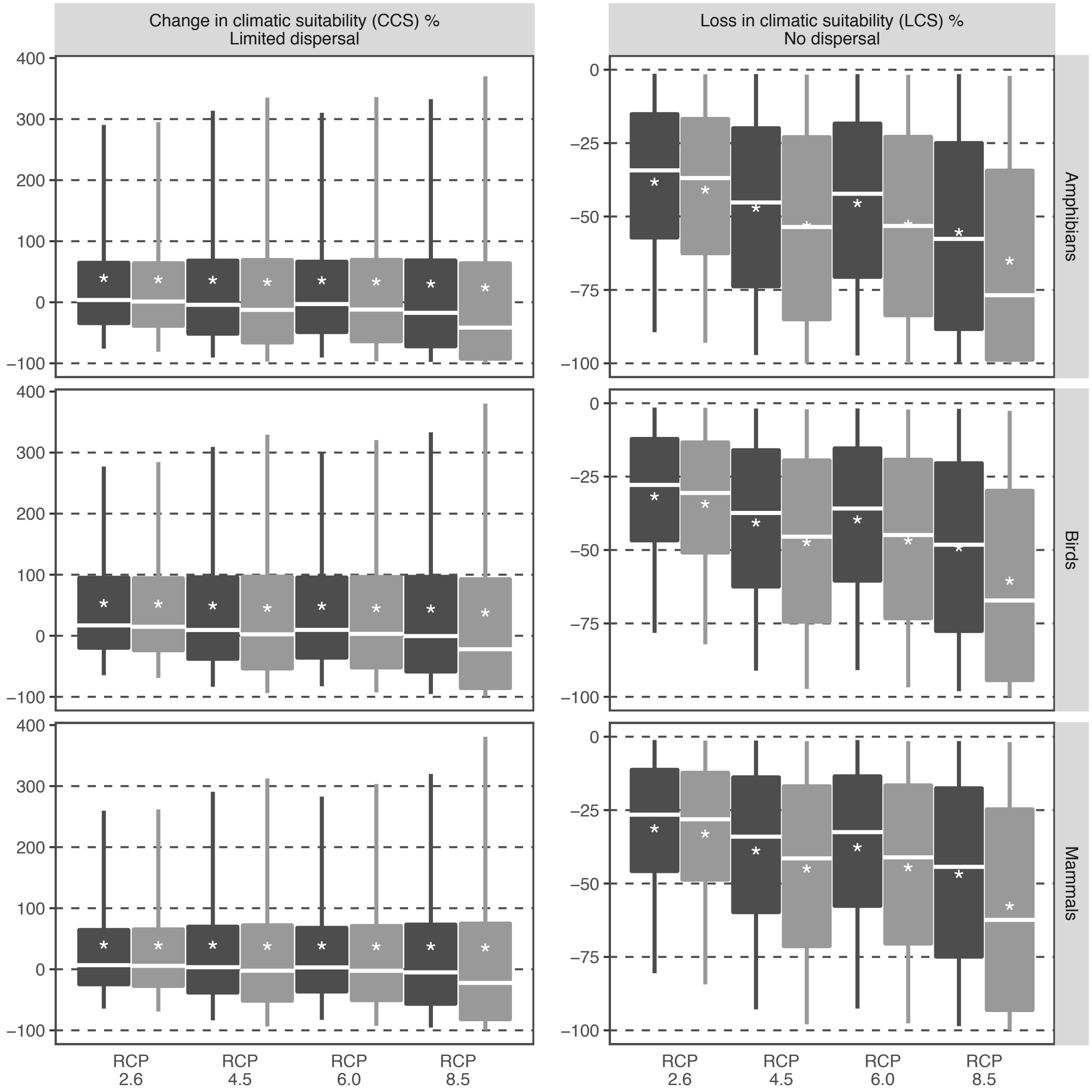

Supplementary Figure 4. Effects of varying the RCP scenarios across a single GCM (IPSL-CM5A-LR) and all SDMs. Dark and clear grey bars represent time horizon 2050 and 2070, respectively.

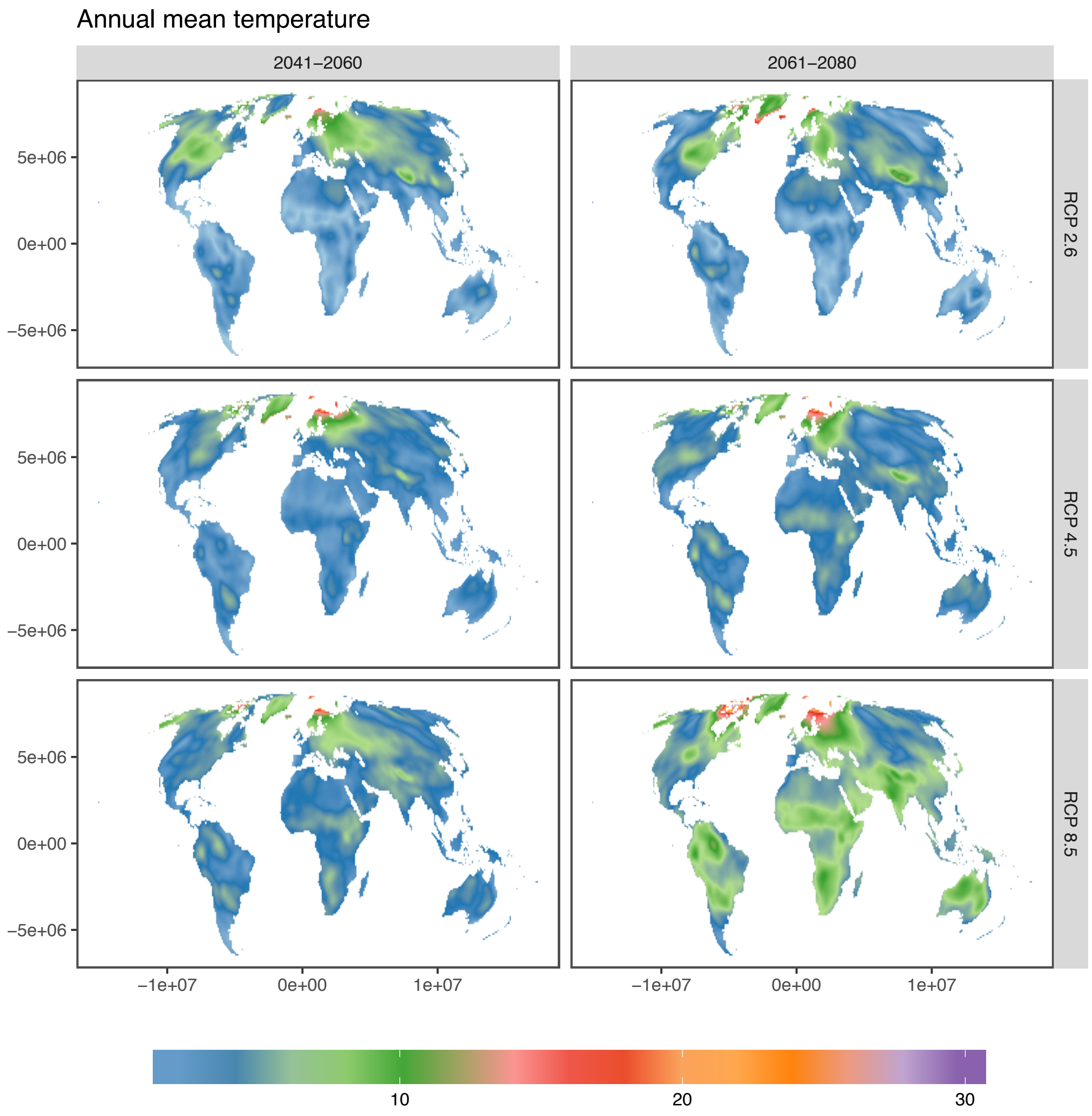

Supplementary Figure 5. Standard deviation of projected annual temperatures across the different five different GCMs for each RCP and time horizon (2050 and 2070).

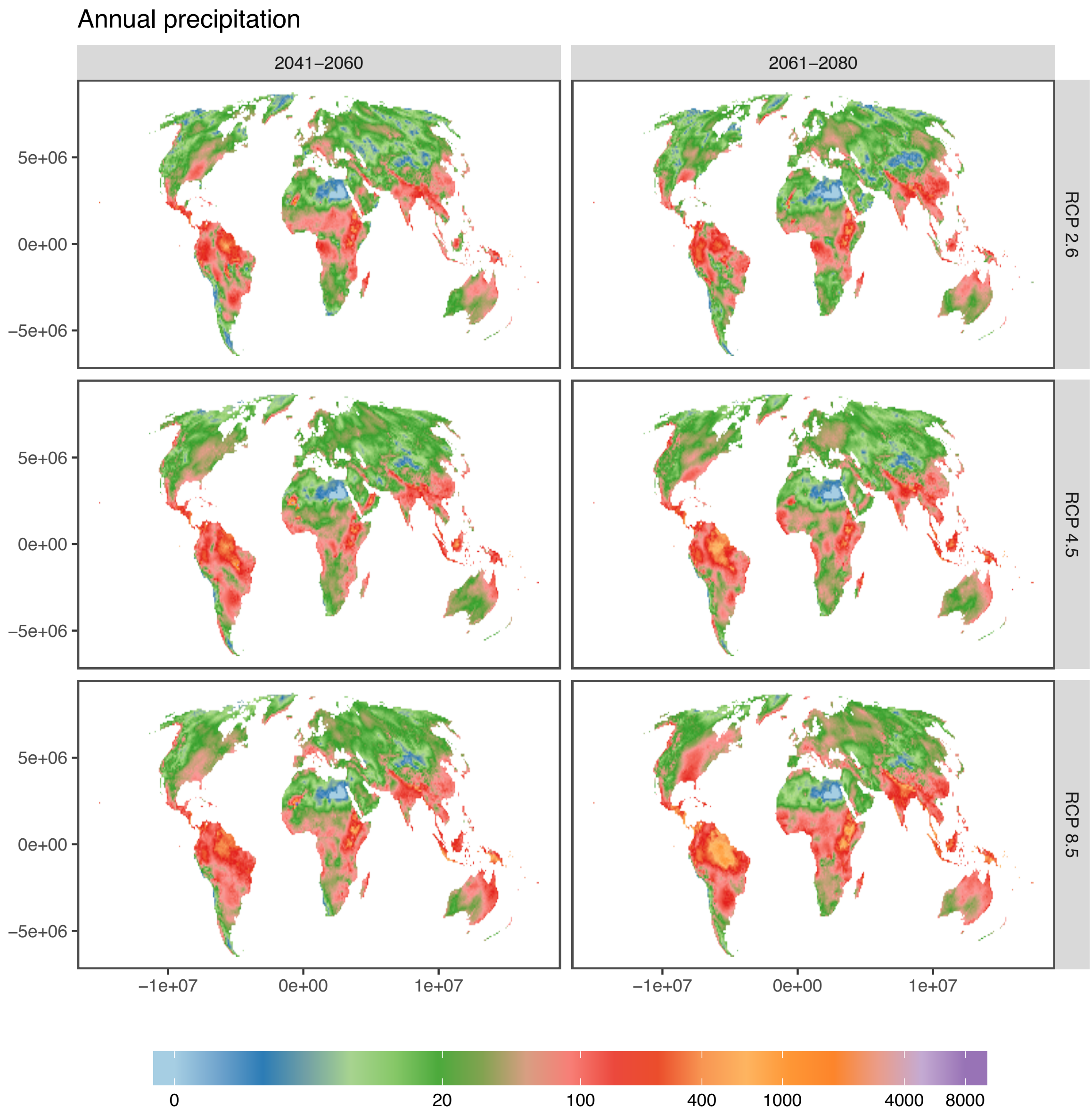

Supplementary Figure 6. Standard deviation of projected sum of precipitations across the different five different GCMs for each RCP and time horizon (2050 and 2070).

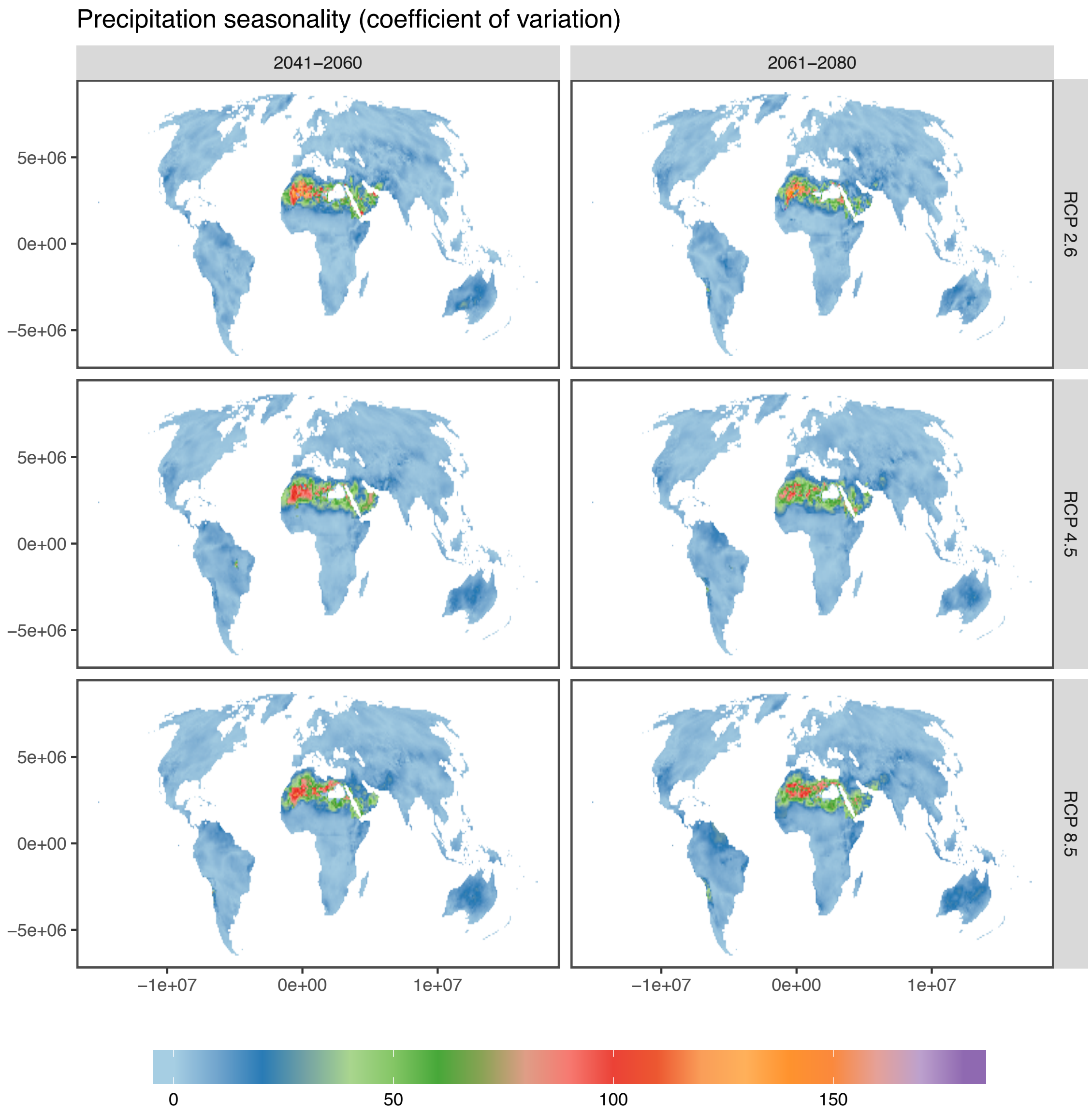

Supplementary Figure 7 Standard deviation of projected precipitation seasonality (coefficient of variation across the year) across the different five different GCMs for each RCP and time horizon (2050 and 2070).

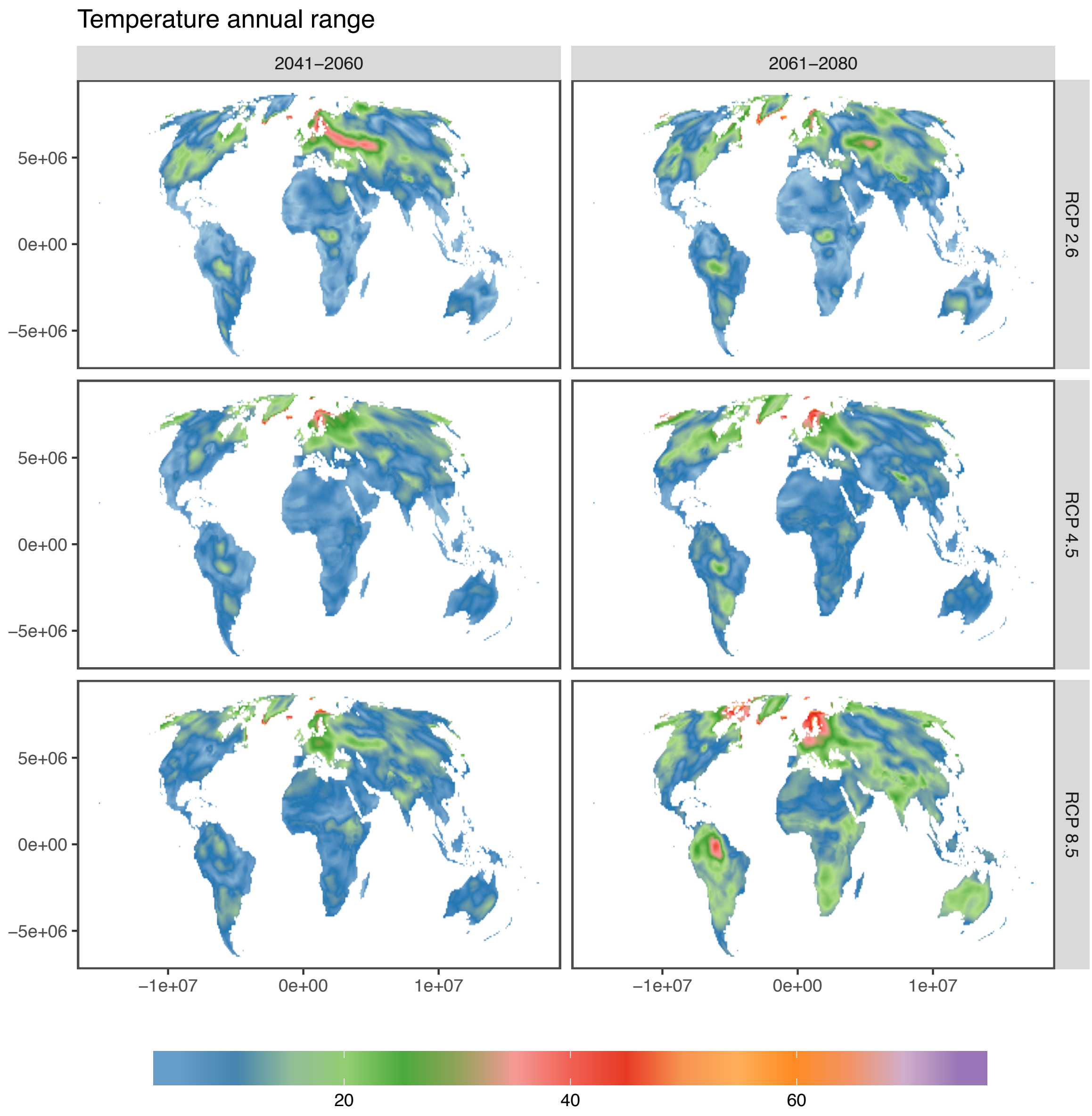

Supplementary Figure 8. Standard deviation of projected annual range in temperatures across the different five different GCMs for each RCP and time horizon (2050 and 2070).

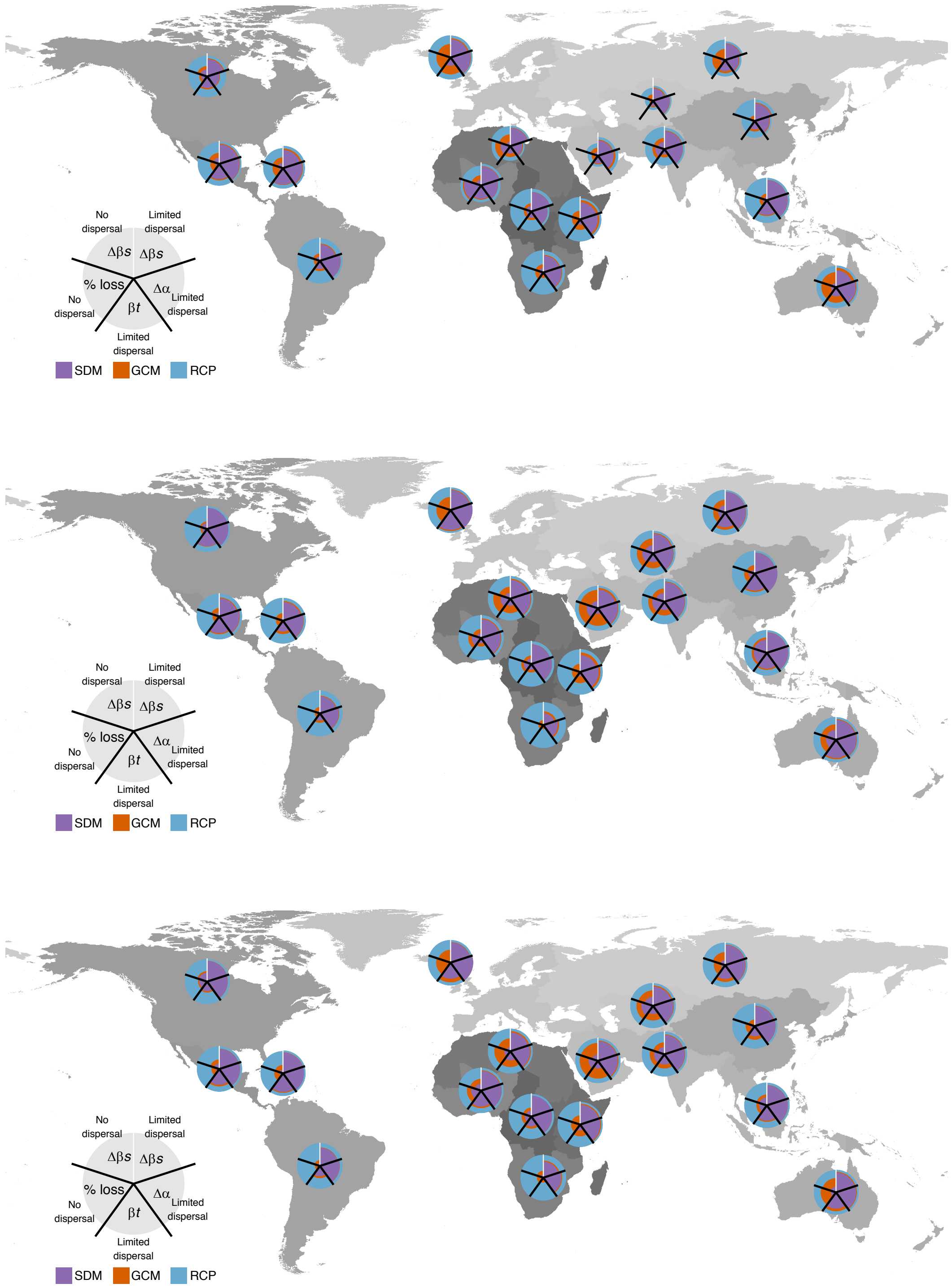

Supplementary Figure 9 : Relative influence (i.e. % of explained variance) of the choice of species distribution models (SDMs), global circulation models (GCMs) and representative concentration pathways (RCPs) on the change in α-diversity per-pixel (Δα), temporal turnover (βt), percentage of species loss per pixel (% loss) and change in β-diversity per IPBES sub-region (Δβs). Results are for the time horizon 2041-2060.

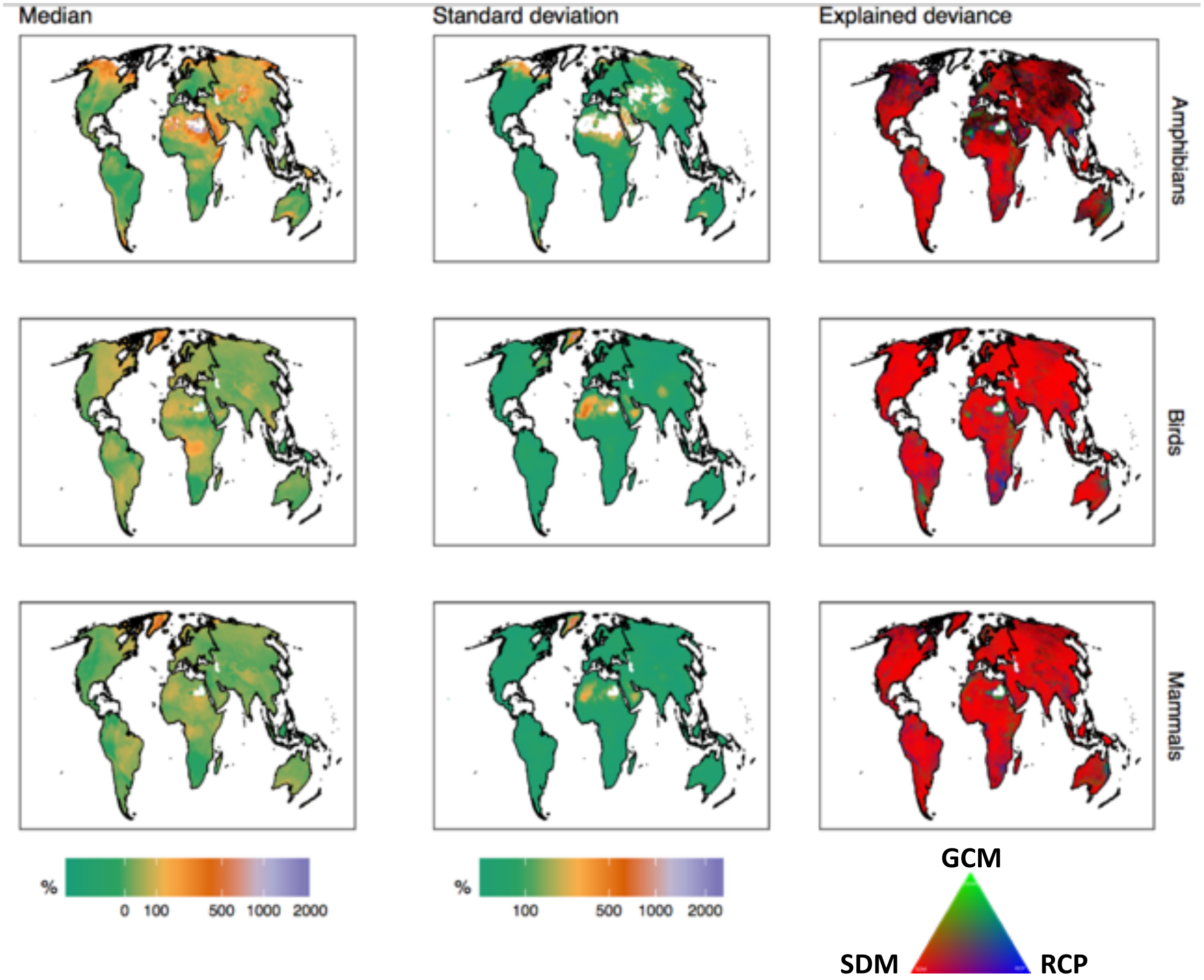

Supplementary Figure 10. Median (left column) and standard deviation (middle column) of change in α-diversity between current and future (2041-2060) climate conditions. Relative importance of SDMs, GCMs, and RCPs in the explained deviance of the ensemble projections (right column). The explained deviance is represented by an RGB-color scheme. The more reddish, greenish, blueish, the more the choice of SDMs, GCMs and RCMs explain the total variation among projections.

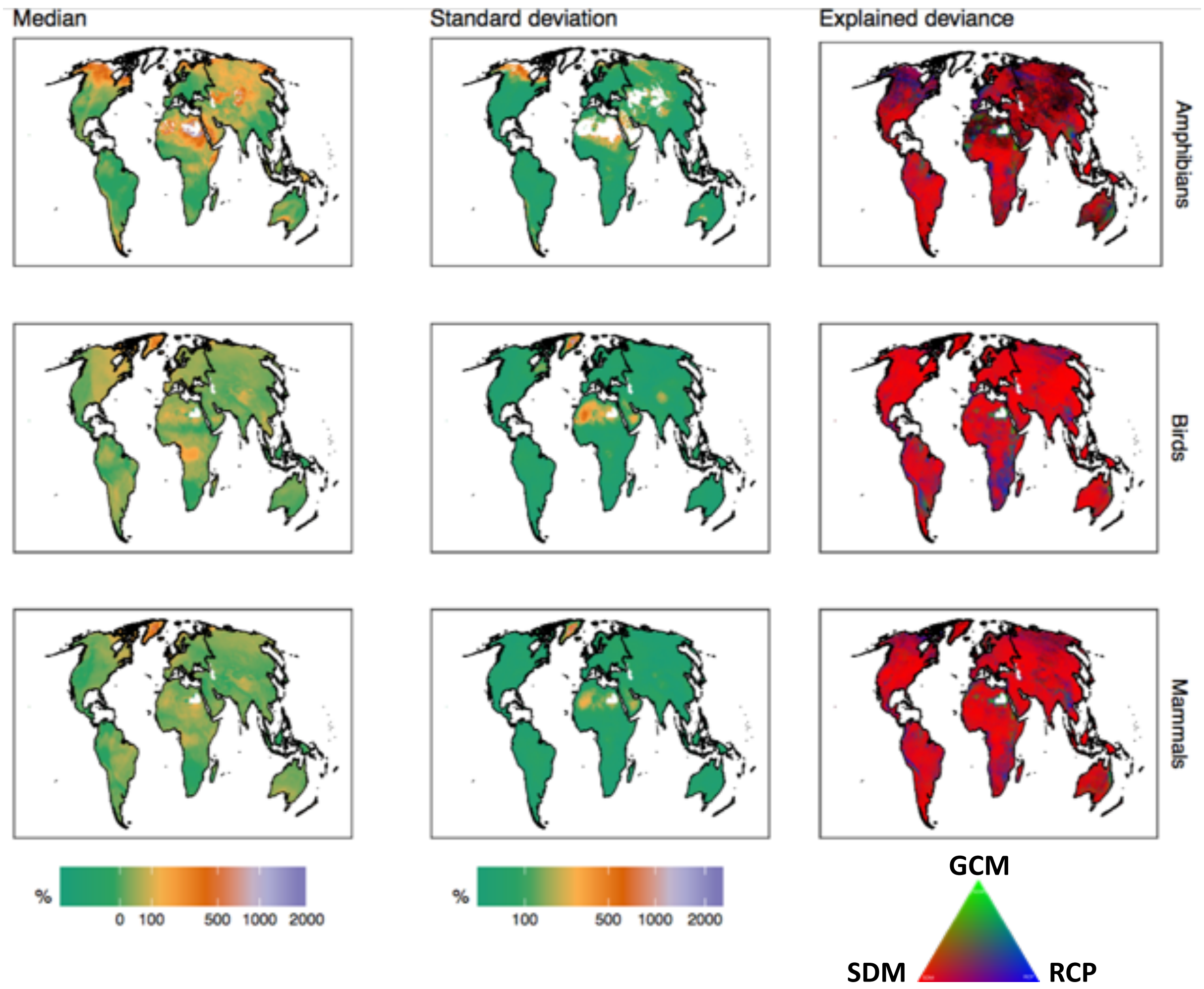

Supplementary Figure 11. Median (left column) and standard deviation (middle column) of change in α-diversity between current and future (2061-2080) climate conditions. Relative importance of SDMs, GCMs, and RCPs in the explained deviance of the ensemble projections (right column). The explained deviance is represented by an RGB-color scheme. The more reddish, greenish, blueish, the more the choice of SDMs, GCMs and RCMs explain the total variation among projections.

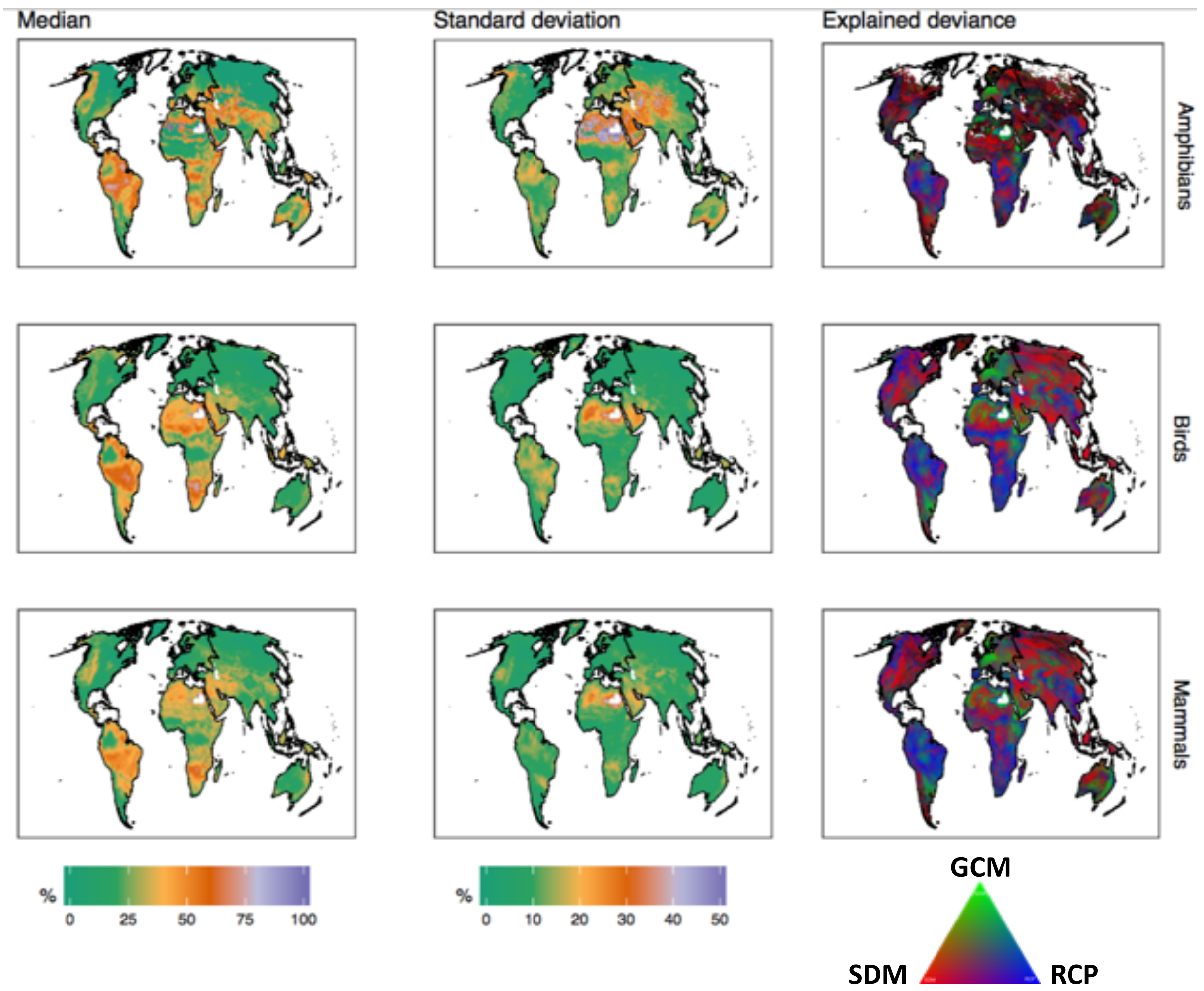

Supplementary Figure 12. Median (left column) and standard deviation (middle column) of percentage of species loss (% loss) per pixel between current and future (2041-2060) climate conditions. Relative importance of SDMs, GCMs, and RCPs in the explained deviance of the ensemble projections (right column). The explained deviance is represented by an RGB-color scheme. The more reddish, greenish, blueish, the more the choice of SDMs, GCMs and RCMs explain the total variation among projections.

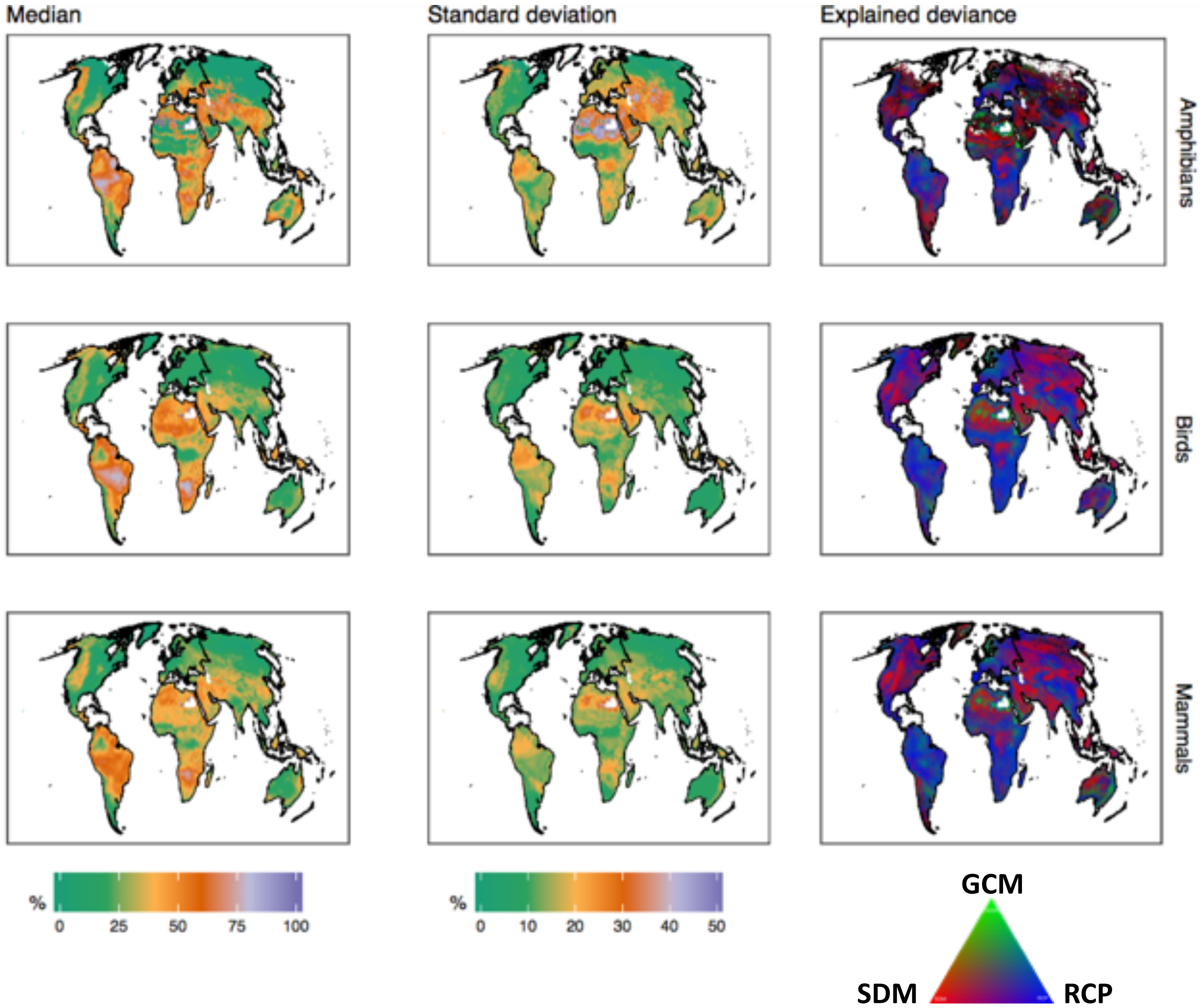

Supplementary Figure 13. Median (left column) and standard deviation (middle column) of percentage of species loss per pixel between current and future (2061-2080) climate conditions. Relative importance of SDMs, GCMs, and RCPs in the explained deviance of the ensemble projections (right column). The explained variance is represented by an RGB-color scheme. The more reddish, greenish, blueish, the more the choice of SDMs, GCMs and RCMs explain the total variation among projections.

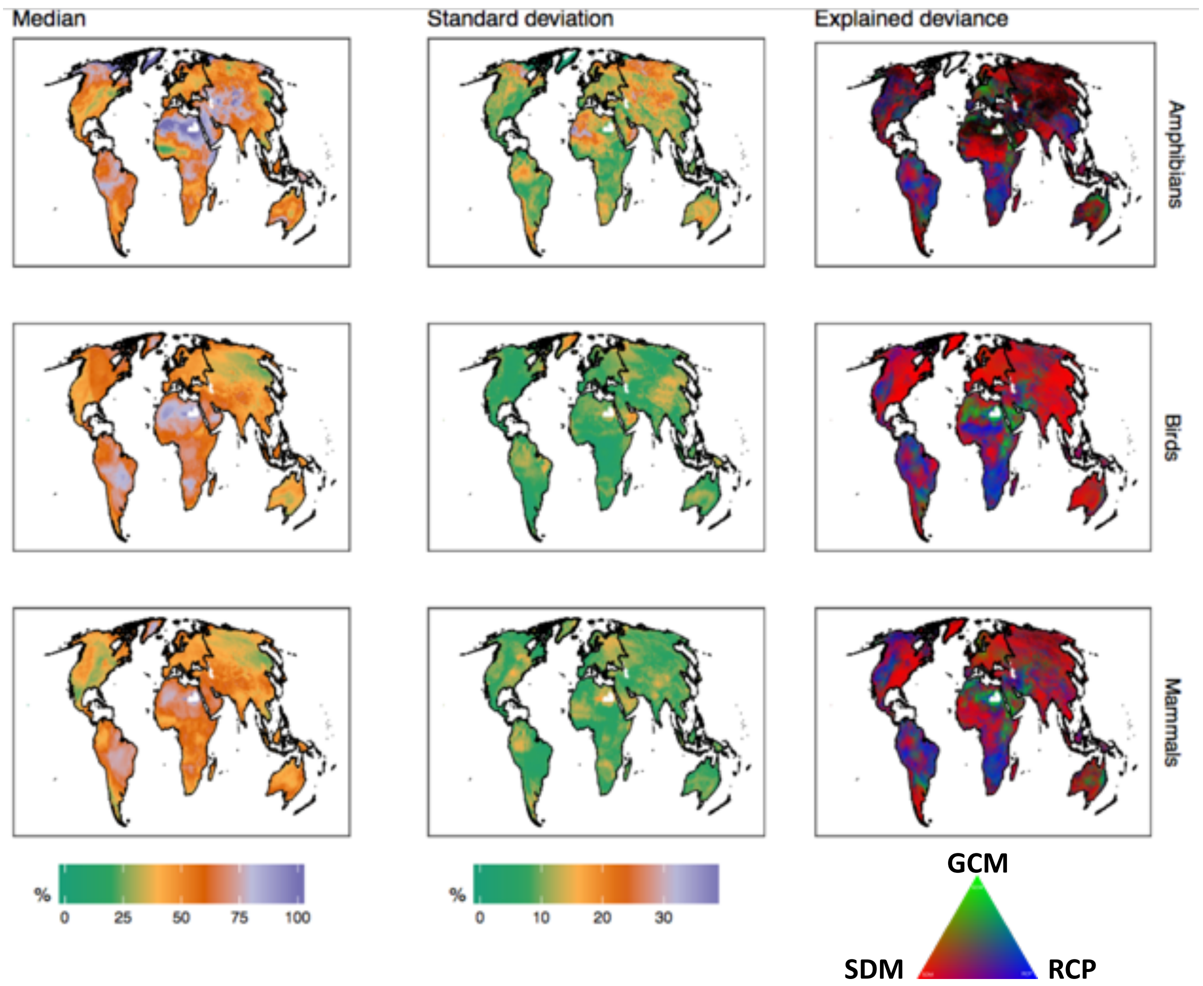

Supplementary Figure 14. Median (left column) and standard deviation (middle column) of temporal species turnover per pixel between current and future climate conditions (2041-2060). Relative importance of SDMs, GCMs, and RCPs in the explained deviance of the ensemble projections (right column). The explained deviance is represented by an RGB-color scheme. The more reddish, greenish, blueish, the more the choice of SDMs, GCMs and RCMs explain the total variation among projections.

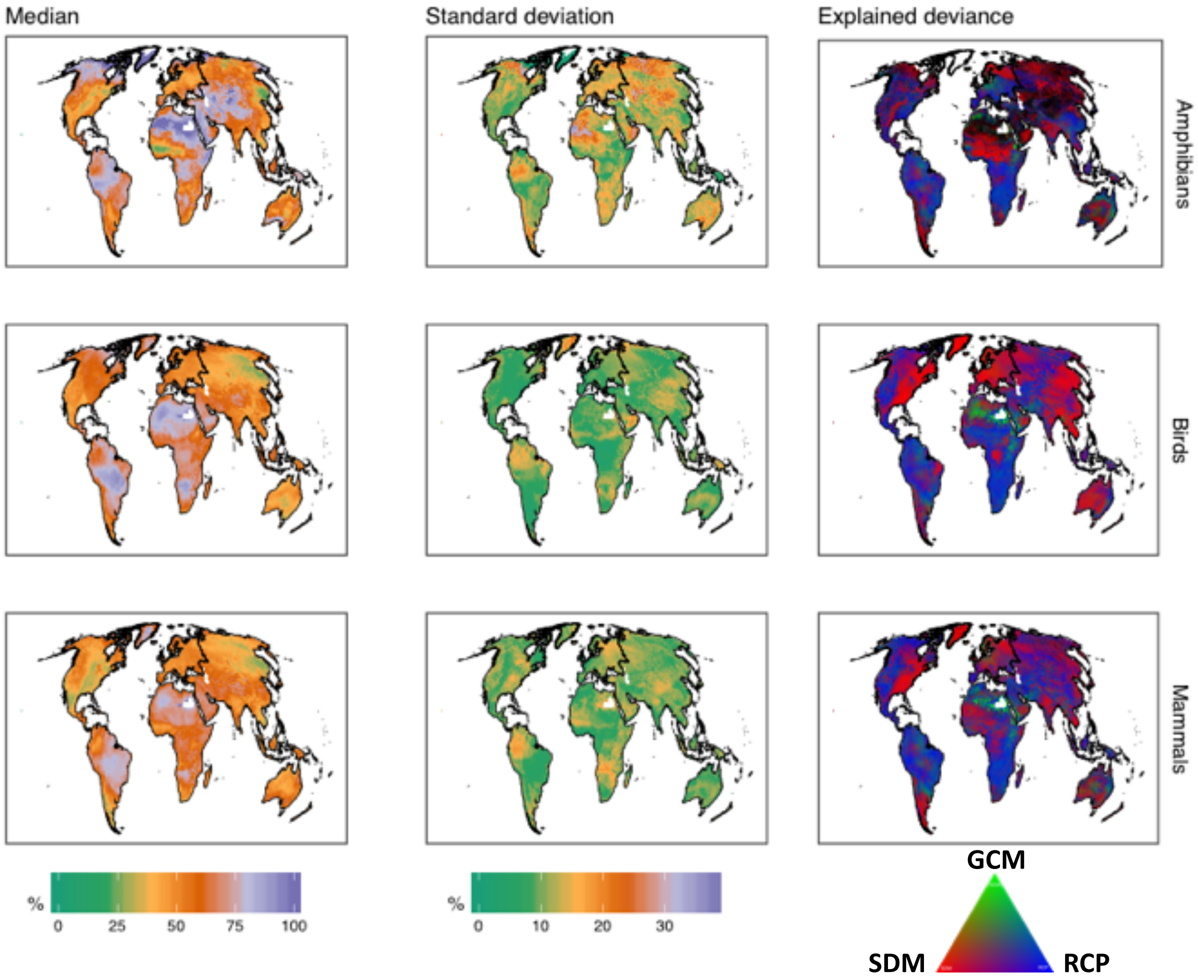

Supplementary Figure 15. Median (left column) and standard deviation (middle column) of temporal species turnover per pixel between current and future climate conditions (2061-2070). Relative importance of SDMs, GCMs, and RCPs in the explained deviance of the ensemble projections (right column). The explained deviance is represented by an RGB-color scheme. The more reddish, greenish, blueish, the more the choice of SDMs, GCMs and RCMs explain the total variation among projections.

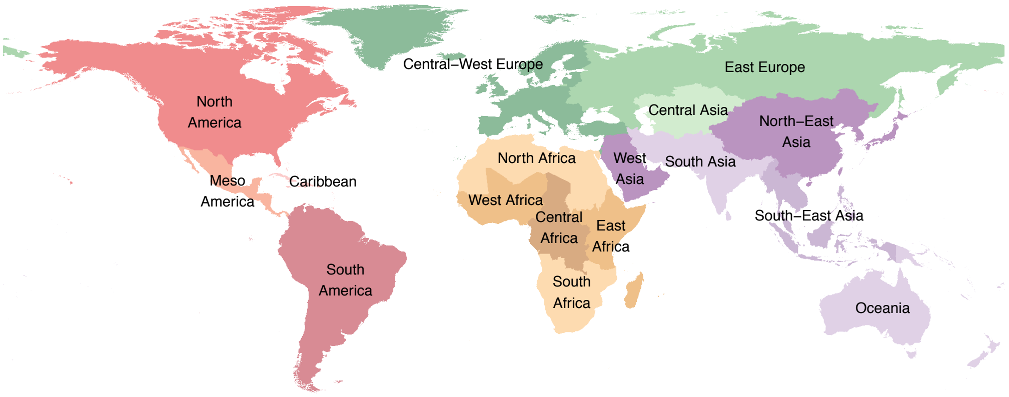

Supplementary Figure 16. Regions and sub-regions defined by the IPBES to communicate results on biodiversity assessment and scenarios. Broad color scheme represents the regions, color ramping within broad color scheme represents sub-regions.
