# Supplementary material for: Uncertainty in ensembles of global biodiversity scenarios
Source: Nat Commun. 2019 Mar 29;10:1446. doi: 10.1038/s41467-019-09519-w (PMC6441032; doi:10.1038/s41467-019-09519-w)
Supplement: Supplementary file 3 — Reporting Summary [file 41467_2019_9519_MOESM3_ESM.pdf]

nature research

Corresponding author(s): Thuiller
Last updated by author(s): Mar 14, 2019

Reporting Summary

Nature Research wishes to improve the reproducibility of the work that we publish. This form provides structure for consistency and transparency in reporting. For further information on Nature Research policies, see Authors & Referees and the Editorial Policy Checklist.

Statistics

For all statistical analyses, confirm that the following items are present in the figure legend, table legend, main text, or Methods section.

n/a	Confirmed	
☐	☒	The exact sample size (*n*) for each experimental group/condition, given as a discrete number and unit of measurement
☒	☐	A statement on whether measurements were taken from distinct samples or whether the same sample was measured repeatedly
☒	☐	The statistical test(s) used AND whether they are one- or two-sided<br>*Only common tests should be described solely by name; describe more complex techniques in the Methods section.*
☐	☒	A description of all covariates tested
☒	☐	A description of any assumptions or corrections, such as tests of normality and adjustment for multiple comparisons
☐	☒	A full description of the statistical parameters including central tendency (e.g. means) or other basic estimates (e.g. regression coefficient) AND variation (e.g. standard deviation) or associated estimates of uncertainty (e.g. confidence intervals)
☐	☒	For null hypothesis testing, the test statistic (e.g. *F*, *t*, *r*) with confidence intervals, effect sizes, degrees of freedom and *P* value noted<br>*Give P values as exact values whenever suitable.*
☒	☐	For Bayesian analysis, information on the choice of priors and Markov chain Monte Carlo settings
☒	☐	For hierarchical and complex designs, identification of the appropriate level for tests and full reporting of outcomes
☒	☐	Estimates of effect sizes (e.g. Cohen's *d*, Pearson's *r*), indicating how they were calculated

Our web collection on statistics for biologists contains articles on many of the points above.

Software and code

Policy information about availability of computer code

Data collection	We used the R software (v 3.5.1.) to format and clean the data downloaded from Data Portals
Data analysis	We use the R software (v 3.5.1.) The code for running all analyses is available on https://gricad-gitlab.univ-grenoble-alpes.fr/leca/publications/thuiller_2019_natcomm.

For manuscripts utilizing custom algorithms or software that are central to the research but not yet described in published literature, software must be made available to editors/reviewers. We strongly encourage code deposition in a community repository (e.g. GitHub). See the Nature Research guidelines for submitting code & software for further information.

Data

Policy information about availability of data

All manuscripts must include a data availability statement. This statement should provide the following information, where applicable:

- Accession codes, unique identifiers, or web links for publicly available datasets
- A list of figures that have associated raw data
- A description of any restrictions on data availability

All data used in this paper are freely available and downloadable from the web. Species distribution maps were provided by the Amphibian and Mammal Red List Assessment (http://www.iucnredlist.org/). For birds, breeding range distribution maps were extracted from BirdLife (http://www.birdlife.org/). All climatic data are available on the CHELSA data portal (http://chelsa-climate.org)

Field-specific reporting

Please select the one below that is the best fit for your research. If you are not sure, read the appropriate sections before making your selection.

☐ Life sciences ☐ Behavioural & social sciences ☒ Ecological, evolutionary & environmental sciences

For a reference copy of the document with all sections, see nature.com/documents/nr-reporting-summary-flat.pdf

Ecological, evolutionary & environmental sciences study design

All studies must disclose on these points even when the disclosure is negative.

Study description	We modelled the global distribution of ~11,500 amphibian, bird and mammal species and project their climatic suitability into the time horizon 2050 and 2070, while varying the input data used. By this, we explore the uncertainties originating from selecting species distribution models (five SDMs), dispersal strategies (two strategies), global circulation models (five GCMs), and representative concentration pathways (four RCPs).
Research sample	The original sample size was 5547, 4616 and 9993 amphibian, mammal and bird species. This represent the whole set of extant species of the world.
Sampling strategy	The data are provided by the IUCN Red List assessment and all the explanations on the sampling strategy is explained, country by country.
Data collection	The data are provided by the IUCN Red List assessment and all the explanations on the sampling strategy is explained, country by country.
Timing and spatial scale	Spatial scale is global. We modelled the distribution of the selected species at 100km resolution under current conditions (1979-2013) and under the selected future periods (2041-2060, 2061-2080).
Data exclusions	We excluded species for which there was not enough presence data to be modelled. We consider 20 presence points the minimum to successfully fit response curves to four different predictor variables. We finally focused on 1,351 amphibian, 7,248 bird and 2,896 mammal species after removing species occurring in less than 20 grid cells, as well as domestic and aquatic species.
Reproducibility	There are no experiments in the study. All statistical analyses are reproducible using the R-code available in the GitLab.
Randomization	There was no randomization procedure. We used a set of four bioclimatic variables from the CHELSA dataset up-scaled from a 1 km to a 100 km resolution. The chosen variables were as follows: annual mean temperature, annual temperature range, annual sum of precipitation and precipitation seasonality (coefficient of variation in monthly sum of precipitations).
Blinding	Blinding was not relevant. The data are provided by the IUCN red list assessment and BirdLife and are standard data in predictive ecology.

Did the study involve field work? ☐ Yes ☒ No

Reporting for specific materials, systems and methods

We require information from authors about some types of materials, experimental systems and methods used in many studies. Here, indicate whether each material, system or method listed is relevant to your study. If you are not sure if a list item applies to your research, read the appropriate section before selecting a response.

Materials & experimental systems

n/a	Involved in the study
☒	☐ Antibodies
☒	☐ Eukaryotic cell lines
☒	☐ Palaeontology
☒	☐ Animals and other organisms
☒	☐ Human research participants
☒	☐ Clinical data

Methods

n/a	Involved in the study
☒	☐ ChIP-seq
☒	☐ Flow cytometry
☒	☐ MRI-based neuroimaging
